# Microscopic mechanisms contributing to the synchronous improvement of strength and plasticity (SISP) for TWIP copper alloys

**DOI:** 10.1038/srep09550

**Published:** 2015-04-01

**Authors:** R. Liu, Z. J. Zhang, L. L. Li, X. H. An, Z. F. Zhang

**Affiliations:** 1 ShenyangNational Laboratory for Materials Science, Institute of Metal Research, Chinese Academy of Sciences, Shenyang 110016, P. R. China

## Abstract

In this study, the concept of “twinning induced plasticity (TWIP) alloys” is broadened, and the underlying intrinsic microscopic mechanisms of the general TWIP effect are intensively explored. For the first aspect, “TWIP copper alloys” was proposed following the concept of “TWIP steels”, as they share essentially the same strengthening and toughening mechanisms. For the second aspect, three intrinsic features of twinning: i.e. “*dynamic development*”, “*planarity*”, as well as “*orientation selectivity*” were derived from the detailed exploration of the deformation behavior in TWIP copper alloys. These features can be considered the microscopic essences of the general “TWIP effect”. Moreover, the effective cooperation between deformation twinning and dislocation slipping in TWIP copper alloys leads to a desirable tendency: the synchronous improvement of strength and plasticity (SISP). This breakthrough against the traditional trade-off relationship, achieved by the general “TWIP effect”, may provide useful strategies for designing high-performance engineering materials.

Strength and plasticity are two sides of the same coin for engineering metallic materials: the improvement of strength generally results in the reduction of plasticity, and vice versa^1^. For years, this trade-off relationship between strength and plasticity has been verified by a large number of experimental results^2^,^3^,^4^,^5^. In fact, this restriction originates essentially from the dislocation mechanism dominating in crystalline materials: the initiation and motion of dislocations, which contribute to plasticity, should be hindered to acquire high strength. In this case, the synchronous improvement of strength and plasticity (SISP) seems to be an impossible mission. However, if we introduce new prevailing strengthening and deformation mechanisms, it would be possible to break through the trade-off relationship between strength and plasticity of metallic materials.

Deformation twinning, as a fundamental deformation mechanism different from dislocation slipping, could be a promising choice. This is exemplified by twinning induced plasticity (TWIP) steels. According to previous investigations^6^,^7^,^8^,^9^,^10^, an outstanding combination of strength and plasticity can be achieved by introducing deformation twinning into single-phase austenitic steels during plastic deformation. To achieve a better understanding and make full use of the TWIP effect, the following two problems should be addressed: (i) is this “TWIP effect” a general mechanism that can be shared by other face centered cubic (FCC) alloys? If so, there should be a “TWIP family” that contains “TWIP steels” and some other “TWIP alloys”. (ii) How does deformation twinning benefit the strength and plasticity simultaneously in “TWIP alloys”?

For the first aspect, because the TWIP effect derives from the behavior of deformation twinning rather than the irreplaceable chemical components of TWIP steels, other FCC metals that can deform by twinning should be evaluated to discover possible similar “TWIP alloys”. Actually, deformation twins have been discovered in various kinds of FCC materials, including alloys with low stacking fault energy (SFE), such as Cu-Al^11^,^12^, Cu-Zn^13^,^14^, Cu-Si^15^,^16^, Cu-Mn^17^, Cu-Ag^18^, and Ni-Fe^19^ and even pure metals with relatively high SFE like Cu^20^, Ni^21^ and Al^22^. As a representative one among them, the Cu-Al alloy has several features similar to those of TWIP steels. Based on our previous investigations^23^,^24^,^25^, for Cu-Al alloys containing relatively high Al content, deformation twinning is a dominant mechanism during conventional plastic deformation procedures. More impressively, the trend of SISP has also been verified experimentally in Cu-Al alloys processed by severe plastic deformation (SPD) and subsequent annealing^12^,^25^, as summarized in Fig. 1. Therefore, it is reasonable that Cu-Al alloys can be considered as the second member of the “TWIP family” in addition to the TWIP steels. As a consequence, Cu-Al alloys are chosen for further investigation in this study.

For the second aspect, previous investigations on TWIP steels^6^,^7^,^8^,^9^,^10^ have already achieved a lot in revealing the role of deformation twins in the TWIP effect. Most of these studies emphasize the outstanding strain-hardening ability of TWIP steels^7^ and attribute this ability to the effective impedance of deformation twins on the gliding dislocations. The effect of deformation twins on strain hardening was firstly proposed by Rémy from his early observation of dislocation pile-up against twin boundaries (TBs)^26^ in the 1970s. Later, Liang *et al.*^27^, Idrissi *et al.*^28^ and Kim *et al.*^29^ confirmed the central contributions of the deformation twinning to the high strain-hardening rate of TWIP steels. These studies considered the deformation twins simply as the ever-increasing obstacles to mobile dislocations, which cause the dynamic Hall-Petch effect during plastic deformation^10^. However, according to the previous research results, TBs can act as either the effective obstacles^30^,^31^,^32^ or the gliding planes of dislocations^22^,^33^, or even the dislocation sources^20^,^34^ in some specific circumstances, resulting in totally different strengthening effects. Therefore, in order to give a better explanation, the fundamental characteristics of deformation twinning should be taken into account. As a classical issue, a large amount of investigations on deformation twinning have been carried out since the 1950s^35^,^36^. The abundant research achievements include the twinning modes, structures of TBs, nucleation and growth processes, as well as mechanistic details such as slip-twin and twin-twin intersections, which draw a whole picture about deformation twinning^37^. Based on these achievements, the unique features of deformation twinning compared with dislocation slipping, as well as the cooperative manner of these two deformation mechanisms, are carefully analyzed in this study in order to reveal the essential mechanisms contributing to the trend of SISP in the “TWIP family” a step further.

## Results

### Basic mechanical properties of pure Cu and Cu-Al alloys

**Figure 1:**
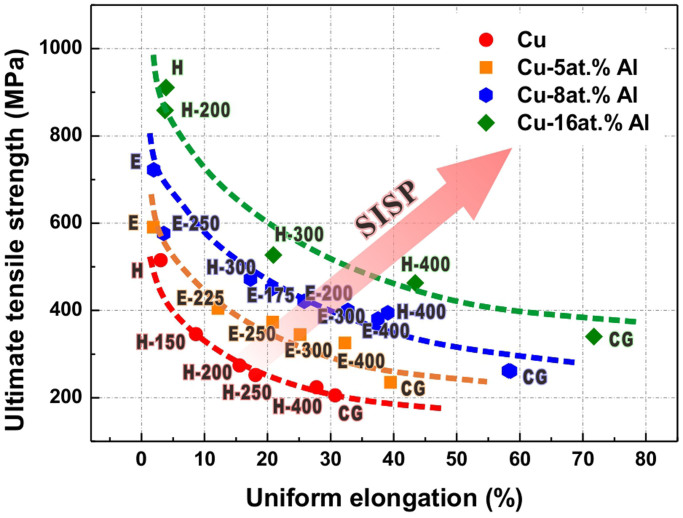
Relationships between UTS and uniform elongation of pure Cu and Cu-Al alloys under different conditions. Cu, Cu-8at.%Al and Cu-16at.%Al after HPT (high pressure torsion, 5 rounds) and subsequent annealing (all for 1 hour)^12^; Cu-5at.%Al and Cu-8at.%Al after ECAP (equal channel angular pressing, 4 passes, route B_C_) and subsequent annealing (all for 1 hour)^25^; characters and numbers beside data points mark corresponding conditions, “H”: HPT, “E”:ECAP, numbers after“-”:annealing temperature.

**Figure 2:**
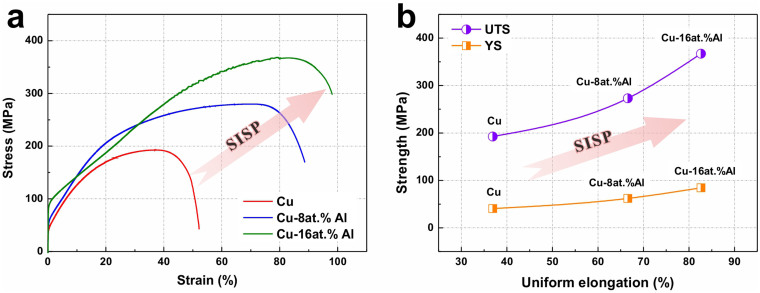
Fundamental mechanical behaviors of pure Cu and Cu-Al alloys during tensile tests. (a) Engineering stress-strain curves. (b) Relationships between ultimate tensile strength (UTS), yield strength (YS, the yield strength at 0.2 percent off-set, *σ*_0.2_) and uniform elongation.

Mechanical properties of pure Cu, Cu-8at.%Al and Cu-16at.%Al alloys during tensile tests are briefly summarized in Fig. 2. The tensile stress-strain curves in Fig. 2a and the relationships between yield strength (YS, the off-set yield at 0.2 percent, *σ*_0.2_), ultimate tensile strength (UTS) and uniform elongation in Fig. 2b show an obvious SISP tendency with increasing Al content. These experimental results, together with our previous findings (summarized in Fig. 1), indicate that the trend of SISP is an intrinsic characteristic of Cu-Al alloys, which is quite different from the traditional trade-off relationship^2^,^3^,^4^,^5^. Moreover, in Fig. 2b, it is worth noting that the increasing tendency of UTS is more obvious than that of YS. While the increase of YS mainly reflects the effect of solid solution strengthening, the extra increase of UTS probably derives from the following work-hardening process, which is possibly associated with the participation of deformation twinning during plastic deformation.

### Typical microstructures of pure Cu and Cu-Al alloys after tensile deformation

**Figure 3:**
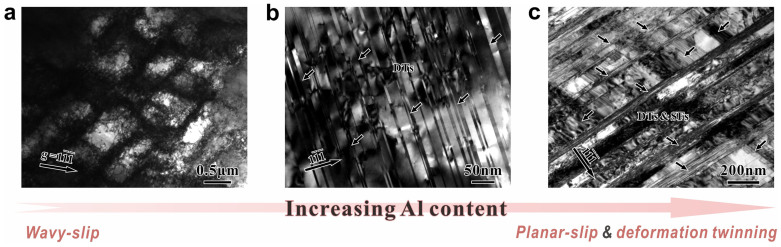
Typical microstructures of pure Cu and Cu-Al alloys observed by TEM after tensile tests. (a) Pure Cu, *e_f_* = 0.34, two-beam bright field image with an observation direction near [110] zone axis, **g** = [1 -1 -1]; (b) Cu-8at.%Al, *e_f_* = 0.49, bright field image along [110] zone axis; (c) Cu-16at.%Al, *e_f_* = 0.63, bright field image along [110] zone axis. The arrow on the bottom illustrates the change of deformation manner with increasing Al content.

As shown in Fig. 3, corresponding to the different mechanical properties of pure Cu and Cu-Al alloys, the deformation mechanisms and microstructure evolution during the tensile tests are also varied. For pure Cu, dislocation cells and bands formed by tangles of dislocations are the typical microstructures (Fig. 3a). In contrast, Cu-8at.%Al shows a mixed structure of planar dislocations and deformation twins (Fig. 3b). As displayed in Fig. 3c, uniform twin lamellas and stacking faults (SFs) are commonly observed in Cu-16at.%Al. The above phenomena can be well explained by the transition of plastic deformation mechanisms with varying SFE. In principle, the decrease of SFE can influence the deformation behavior in the following two aspects: on the one hand, it reduces the possibility of cross slip and thus leads to the transition of dislocation slip from wavy slip to planar slip^25^; on the other hand, it decreases the critical stress of deformation twinning, making it a feasible deformation mechanism during regular deformation^12^. Since there is a notable decrease of SFE in Cu-Al alloys with increasing Al content^38^,^39^, the dominant deformation mechanisms will change gradually. Wavy slip is the most common deformation manner for pure Cu, yet plastic deformation is conducted by the collaboration of planar slip and deformation twinning for Cu-8at.%Al and Cu-16at.%Al alloys. According to the above experimental results and analysis, the observed microstructures in pure Cu and Cu-Al alloys are consistent with the transition of deformation mechanisms, as summarized in Fig. 3.

### Concept of TWIP copper alloys: the correlation between deformation mechanisms and mechanical properties

**Figure 4:**
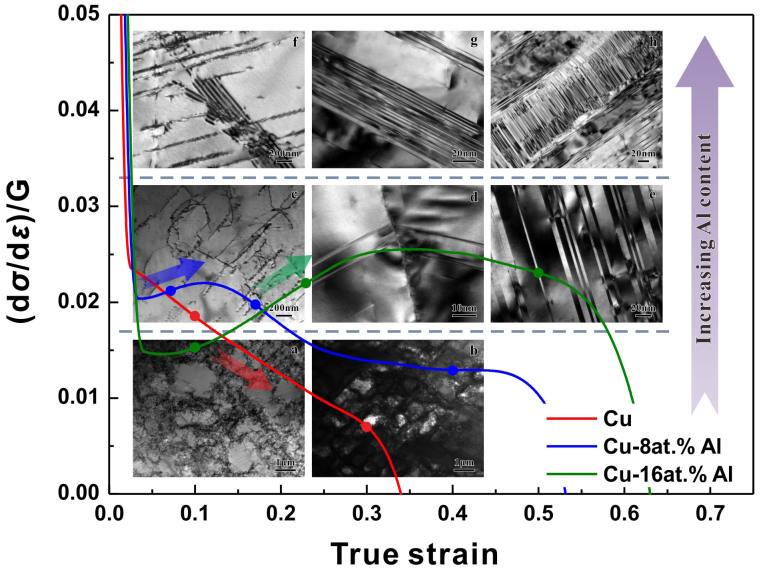
Strain hardening behaviors and corresponding microstructure evolution processes. The large figure: strain hardening rate curves ((d*σ*/d*ε*)/*G*-*e*) of pure Cu and Cu-Al alloys, obtained from the tensile tests. The small pictures: representative microstructures at various strains (corresponding to the marked points on curves) observed by TEM. (a) – (b) Pure Cu: (a) the tangle dislocations and unformed dislocation cells, *e* = 0.10; (b) dislocation cells, *e* = 0.30. (c) – (e) Cu-8at.%Al: (c) dislocations slipping in planar way, *e* = 0.06; (d) deformation twins nucleated on GB, *e* = 0.17; (e) deformation twin lamellas, *e* = 0.40. (f) – (h) Cu-16at.%Al: (f) stacking faults and dislocations slipping in planar way, *e* = 0.10; (g) deformation twin bundles, *e* = 0.23; (h) two fold deformation twins, *e* = 0.50.

The above results have verified the differences in both the mechanical properties and deformation mechanisms between pure Cu and Cu-Al alloys. Therefore, the following investigations and discussions will concentrate mainly on the connections between the two aspects. As shown in Fig. 4, the representative microstructures at different tensile strains and the trends of corresponding strain hardening curves embody their consistency with each other. In the initial stage, large amounts of dislocation tangles spread all over the interior of the grains of pure Cu (Fig. 4a) and bring about a relatively high strain hardening rate (Θ = (d*σ*/d*ε*)/*G*). In the Cu-8at.%Al alloy, dislocations distribute on parallel slip planes in order (Fig. 4c), resulting in lower strain-hardening rate in comparison with pure Cu. For the Cu-16at.%Al alloy, the weak interaction between planar dislocations and SFs (Fig. 4f) leads to further decrease of the initial strain-hardening rate. However, in the following deformation process, dislocations in Cu can quickly form relatively stable structures like cells and walls (Fig. 4b) due to the high recovery rate, leading to a steep drop of strain-hardening rate. In contrast, the strain-hardening rates of the Cu-8at.%Al and Cu-16at.%Al alloys begin to increase and soon exceed the straight declining curve of pure Cu. This may be associated with the low recovery rate of planar dislocations and the emergence of deformation twins (see Fig. 4d, e and g). Moreover, in the later stage of plastic deformation, more intensively distributed twin lamellas (Fig. 4g) and the two or three fold deformation twins formed (Fig. 4h) in the Cu-16at.%Al alloy play an important role in the more remarkable and long-lasting strain-hardening process compared with the Cu-8at.%Al alloy (Fig. 4e). Interestingly, during tensile deformation the nucleation and propagation of deformation twins appear to directly correlate with the upward trend in the strain-hardening rate.

The above results show a close relationship between the mechanical properties and the deformation mechanisms. With the increase of Al content, wavy slip is gradually replaced by planar slip and deformation twinning (Fig. 3), along with the increasing strain hardening efficiency (Fig. 4) and the SISP tendency (Fig. 2). In addition, the detailed comparisons between the nucleation and propagation processes of deformation twins and the strain-hardening behaviors also reveal a good agreement (Fig. 4). Therefore, deformation twinning in Cu-Al alloys probably plays an irreplaceable role in improving the mechanical properties. Moreover, by comparing Cu-Al alloys with TWIP steels, we can easily find that they share similar deformation mechanisms^27^, similar characteristics of deformation twinning^40^, and almost the same tendency of improving mechanical properties^7^. In this scenario, the concept of “TWIP copper alloys” can be naturally proposed with two common principles: (i) deformation twinning acts as a major deformation mechanism; (ii) deformation twinning significantly improves the mechanical properties.

It is crucial to notice that the SISP tendency has been widely discovered in some other alloy systems such as Cu-Zn^13^, Cu-Mn^17^ and Ni-Fe^19^, with the similar transformation of plastic deformation mechanisms. Therefore, it is reasonable to infer that the general improvement in mechanical properties is probably a reflection of a uniform rule that affects mechanical behaviors via deformation mechanisms. This uniform rule, named the “general TWIP effect”, can be shared by a wide range of “TWIP alloys” briefly summarized as the “TWIP family”.

## Discussion

In this part, the underlying intrinsic microscopic mechanisms contributing to the promising SISP tendency in TWIP copper alloys will be analyzed in detail, which are associated with the features of deformation twinning.

### Features of deformation twinning in TWIP copper alloys

**Figure 5:**
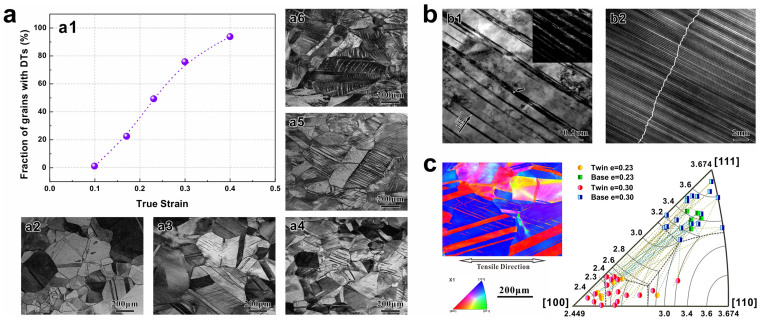
Features of deformation twinning in TWIP copper alloys. (a) Dynamic evolution processes of deformation twinning in Cu-8at.%Al alloy. (a1) Fraction of grains containing deformation twins as a function of true strain; the data were obtained from following LEXT observations. (a2) – (a6) Typical microstructures observed by LEXT at different strains: (a2) *e* = 0.10; (a3) *e* = 0.17; (a4) *e* = 0.23; (a5) *e* = 0.30; (a6) *e* = 0.40. (b) Typical microstructure of deformation twins in TWIP copper alloys observed in Cu-16at.%Al after tensile failure (*e_f_* = 0.63). (b1) TEM micrographs of twin bundles in bright field pattern and corresponding dark field image, observed along [110] zone axis. (b2) HRTEM image of the region marked in b1, taken along [110] zone axis; the K_2_ planes are marked by the white broken line across the matrix, deformation twins and SFs. (c) Microstructure of deformation twins and the grains in Cu-8at.%Al at true strain of 0.30 observed by SEM-EBSD, with data clean-ups performed (left); and statistical results about orientation of deformation twins and corresponding bases in Cu-8at.%Al at true strains of 0.23 and 0.30, with an isoline distribution of the Taylor factor as background of the inverse pole figure (right).

To achieve a more in-depth comprehension of the microscopic mechanisms leading to the TWIP effects, it is necessary to investigate the characteristics of deformation twinning. As summarized in Fig. 5 in this section, three features of deformation twinning in TWIP copper alloys will be discussed in comparison with the common dislocation slipping process.

The first feature can be concluded as “***dynamic development***”. In TWIP copper alloys, twinning actually acts as a gradually and commonly developed deformation mode. Fig. 5a shows this process clearly: the statistics in Fig. 5a-a1 displays the proportions of grains in which deformation twins can be observed by LEXT (a typical laser confocal scanning microscope). Fig. 5a-a2 to a6 show typical microstructures observed by LEXT at each strain corresponding to the five data in Fig. 5a-a1. According to these figures, for the Cu-8at.%Al alloy, deformation twinning rarely occurs until the true strain increases to 0.10 (see Fig. 5a-a2). With the continuously increasing strain, deformation twins nucleate gradually either at grain boundaries (GBs) or at annealing TBs, and expand rapidly into the interior of the grains (see Fig. 5a-a3 to a5). When the true strain rises to 0.40, deformation twin bundles can be observed in more than 90% of the grains (see Fig. 5a-a1 and a6) and the twins would probably continue propagating with increasing strain. In short, the deformation twins gradually develop during plastic deformation and can ultimately occur in most grains in TWIP copper alloys.

The second feature can be described by the key word “***planarity***”. Fig. 5b displays the most common microstructure of deformation twins in TWIP copper alloys observed by transmission electron microscopy (TEM) and high resolution transmission electron microscopy (HRTEM). The dark lines shown in Fig. 5b-b1 (the bright lines in corresponding dark field photo in the top right corner) are usually called twin bundles, because each bundle actually contains a certain amount of twin lamellas and SFs, as displayed in Fig. 5b-b2. Deformation twin lamellas in each bundle are nano-scaled in the thickness direction, in contrast with the much larger size in the length directions. During the deformation process, twinning usually prefers to develop by producing new twin lamellas in a bundle, rather than broadening the existing twin lamellas. In fact, under a specific deformation condition, the average thickness of twin lamellas is mainly determined by the nature of materials^23^,^24^, such as the value of SFE. According to our research, the twin lamellas of the Cu-8at.%Al alloy with moderate SFE are generally thicker than those of the Cu-16at.%Al alloy with rather low SFE. In comparison with dislocation slipping, the deformation mode and structural characteristic of twinning are both more planar, making it possible to form the nano-scaled lamellas which are in a much smaller dimension than the common dislocation configurations.

The last feature can be summarized as “***orientation selectivity***”. According to Fig. 5a, deformation twinning usually starts in several grains and then disperses to others. With the help of electron backscattered diffraction (EBSD), the orientation information of the grains wherein deformation twins emerge at early stages (the true strain *e* = 0.23, 0.30) is displayed in Fig. 5c. According to the inverse pole figure on the right side of Fig. 5c, for the most common condition (statistics in tensile direction), deformation twinning would prefer to nucleate in grains with orientations near <111> (with Taylor factors larger than 3.1, referring to background of the figure); corresponding twin bundles' orientations scatter around <001> (sharing Taylor factors below 2.8). A typical condition is displayed in the EBSD map on the left side. Hence, it can be deduced that deformation twins usually nucleate preferentially in grains with “harder” orientations for slipping, and produce twin bundles with “softer” orientations. Accordingly, it is reasonable to postulate that twinning and slipping are collaborative deformation mechanisms which are complementary to each other in the TWIP copper alloys. In fact, this phenomenon also exists in TWIP steels according to previous research^40^,^41^,^42^.

The above analysis is a brief description of the three features associated with deformation twinning in TWIP copper alloys. In fact, each of the features participates in the microscopic mechanisms of the general TWIP effect, which will be further discussed in the following section.

### Source of strength and plasticity: microscopic mechanisms of TWIP effect

**Figure 6:**
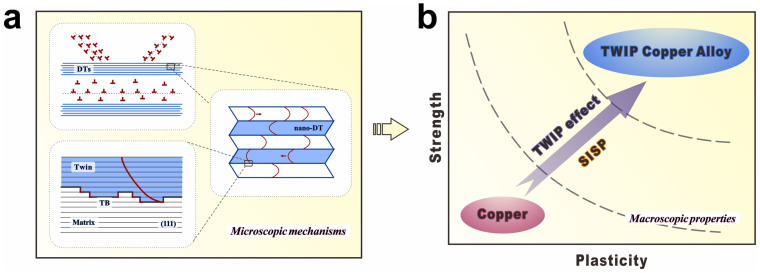
Summarization of microscopic mechanisms of the TWIP effect and corresponding macroscopic properties. (a) Illustrations of the cooperation between deformation twinning and dislocation slipping at three different length scales. (b) The tendency of SISP obtained through TWIP effect.

The tendency of SISP consists of the enhancement of strength and the improvement of plasticity. For TWIP copper alloys, both aspects are the reflections of the cooperation between dislocation slipping and deformation twinning. Corresponding microscopic mechanisms and macroscopic properties are illustrated in Fig. 6. The improved strength should be mainly attributed to the following several aspects.

- **The inhibition of dislocation motion by dynamically produced twin bundles**. During the plastic deformation process, the gradually propagated deformation twins separate the grains into two kinds of sections: the twin bundles and the matrix between them. Since most of the dislocations exist in the matrix, twin lamellas can act as effective obstacles to impede the motion of dislocations, especially for those with slip systems unparallel to the twin lamellas. With increasing plastic strain, the size of the matrix between twin bundles continuously decreases with the increasing propagation range of deformation twins, making this dynamic Hall-Petch effect more remarkable.
- **The strengthening effect of nano-twins.** It is important to notice that the condition in twin bundles is different from that in the matrix between the bundles due to the nano-scale thickness of the twin lamellas. According to the research results of You *et al.*^43^, the special deformation patterns of these nano-twin structures help to improve the strength of twin bundles, which makes the nano-lamellas a kind of “hard phase” in grains.
- **The increasing capacity of defects.** For dislocations in TWIP copper alloys, besides the increased resistance to cross-slip induced by the decrease of SFE, the twin bundles also help to hinder the dislocation recovery by separating them into smaller regions. Moreover, recent studies have indicated that dislocations on TBs tend to be more stable than those in the perfect crystal. Thus, dislocations near the twin lamellas should prefer to gather on TBs^44^. This “absorption” effect markedly improves the twin lamellas' capacity of dislocations. In addition, for deformation twins, it is more difficult than for dislocations to recover (de-twinning) or reach a saturation state due to its planar deformation mode and stable nano-sized structure. In comparison with the wavy-slip of dislocations, the continuous propagation of planar dislocations and deformation twins can lead to higher density of defects in the deformed TWIP copper alloys, along with a more remarkable strengthening effect.

Corresponding to the above strengthening mechanisms, the improved plasticity could be attributed to several factors listed as follows.

- **The outstanding strain-hardening ability.** Compared to pure Cu, the strain-hardening potential of TWIP copper alloys is notably improved by the participation of deformation twinning. The enhanced strain-hardening ability improves the deformation homogeneity, delays the occurrence of necking, and further contributes to a larger uniform elongation. It is worth noting that the outstanding strain-hardening ability of TWIP copper alloys is also an important factor that combines the improved strength and enlarged plasticity.
- **The gliding “tunnel” in lamellar structure.** Even though the existence of deformation twins hinders the motion of dislocations gliding unparallel to the twin lamellas, some relatively “soft” directions still remain. In spaces between neighboring twin bundles, dislocations can slip on planes parallel to TBs without additional resistance. These directions act just like “tunnels” for dislocations. In the nano-scale twin lamellas, these directions are also “softer” than others. These gliding “tunnels” in lamellar structures make it possible for TWIP copper alloys to deform continuously.
- **The extra choice of deformation mechanisms.** Besides acting as the producer of the lamellar defects, deformation twinning is also one of the fundamental mechanisms of plastic deformation. Thus, the twinning behavior per se can contribute to the total plastic strain. As deformation twinning shares the same {111} planes but different directions of <112> compared with dislocation slipping (which usually deforms in directions of <110>) in FCC structure^45^, grains in relatively “hard” orientations for slipping can choose to deform by twinning, as shown in Fig. 5c. The combination of slipping and twinning provides grains more choices to deform compatibly with each other, which is beneficial to the deformation homogenization.

According to the above experimental results and corresponding analysis, it can be confirmed that the general TWIP effect, which is based on the twinning-participated microscopic mechanisms, leads to the SISP trend in TWIP copper alloys, as shown in Fig. 6. The special features of deformation twinning and its effective cooperation with dislocation slipping work together, changing the trade-off relationship between strength and plasticity into a synchronous one. The intrinsic microscopic mechanisms contributing to the SISP tendency can be considered as the essences of the general TWIP effect, which can be shared by the whole “TWIP family”, including TWIP steels and the newly proposed “TWIP copper alloys”.

In summary, we tried to break through two limitations about the theme of “TWIP” in this study: one is the limited alloy systems which sustain the TWIP mechanism; the other is the limited investigations on the microscopic mechanisms underlying the general “TWIP effect”. For the first aspect, a new concept of “TWIP copper alloys” is proposed based on the similar mechanical behaviors and deformation mechanisms between TWIP steels and Cu-Al alloys. In addition to TWIP steels, the TWIP copper alloys can be considered as another member of the “TWIP family”. Following this way, further broadening of the family could also be possible. For the second aspect, the investigations of this work focus on the underlying microscopic mechanisms corresponding to the macroscopic mechanical behaviors. Three microscopic features of deformation twinning were derived from the detailed exploration of the twinning behavior in the proposed TWIP copper alloys, i.e. ***“dynamic development”***, ***“planarity”***, as well as ***“orientation selectivity”***, which can be considered as the microscopic essences of the general “TWIP effect”. The special features of deformation twinning and its effective cooperation with dislocation slipping lead to the SISP tendency. The breakthrough against the traditional trade-off relationship between strength and plasticity suggests new possibilities to design high-performance engineering materials. It is important to seek more “TWIP alloys” and to investigate the general TWIP effect further, for both the fundamental understandings and engineering applications of metallic materials.

## Methods

### Materials fabrication

In this study, we choose two Cu-Al alloys in compositions of Cu-8at.%Al and Cu-16at.%Al, with pure Cu (99.97 wt.%) as a contrast. The Cu-Al alloys were prepared by melting and casting the 99.99 wt.% pure Cu and 99.99 wt.% pure Al. The as-cast pure Cu and Cu-Al alloys were cold-rolled into 4 mm thick plates, following the standard size and process of GB/T 2040-2002 (a Chinese National Standard for sheet of copper and copper alloys). Before tensile tests, the materials were annealed at 800°C for 2 h to diminish the defects produced by rolling and obtain highly homogeneous microstructures with an average grain size of ~150 μm. As the annealed grains of the specimens have typical equiaxed grains, the average grain size was calculated by a so-called “line transversal method”: draw lines in random directions and random locations on pictures obtained by LEXT, and calculate the value of the total length of the lines divided by the total number of grains (including annealing twins) those lines stretched across; the quotient is the approximate value of the average grain size. To make the data credible, about 500 grains were measured for each kind of materials.

### Tensile test

The tensile specimens were cut from the cold-rolled and annealed sheets by a wire cutting machine into a dog-bone shape, which have gauge dimensions of 17 mm × 2 mm × 4 mm, with the tensile axis parallel to the rolling direction. Uniaxial tensile tests were carried out on an AG-X-10KN tensile instrument at a strain rate of 1 × 10^−3^ s^−1^. To obtain the whole tensile stress-strain curve, several specimens were loaded to failure. Each test was repeated at least 3 times to ensure the results were reliable. The other specimens were deformed to specific strains, such as 0.17, 0.23, 0.30, 0.40 *etc*, in order to investigate the processes of plastic deformation. The strain was measured by an extensometer throughout the tensile test.

### Microstructure characterization

The LEXT OLS4000, a laser confocal microscope, was employed to characterize the propagation process and distribution condition of the deformation twins. As the LEXT allows high-definition imaging (resolutions of 0.12 μm on X-Y plane and 20 nm in Z direction are available), it is more effective to observe deformation twins than optical microscopy. The tensile specimens were mechanical polished and then etched by a solution with the composition of FeCl_3_ : HCl : C_2_H_5_OH : H_2_O = 8 g:10 ml : 50 ml : 50 ml, to distinguish the bundles of deformation twins from slip bands. The proportions of grains with deformation twins were obtained by counting more than 300 grains for each specimen.

Electron backscatter diffraction (EBSD) observations were carried out on a LEO Supra 35 field emission scanning electron microscope (SEM), to obtain the accurate orientation information of the deformation twins and corresponding matrixes. Main operating parameters for the SEM and EBSD examinations are listed as follows: the operating voltage is 20 kV, the step size of EBSD is 0.05 μm, and the clean-ups of the EBSD data were performed to diminish the point of zero solutions. The specimens were prepared by mechanical polishing and electro-polishing in a solution of H_3_PO_4_: C_2_H_5_OH : H_2_O = 1:1:2 (vol.) with a voltage of 2–3 V at room temperature.

The details of the deformed microstructures were characterized by transmission electron microscopy (TEM) with an FEI Tecnai F20 microscope, operated at 200 kV. Thin foils for TEM observations were first cut from the tensile specimens parallel to the tensile axis by a wire cutting machine, with an original thickness of 300 μm. Then they were mechanically polished to about 50 μm thick, followed by twin-jet polishing in a solution of H_3_PO_4 _: C_2_H_5_OH : H_2_O = 1:1:2 (vol.) with a voltage of 8–10 V at −6°C. For all the above microstructure characterization methods, the cross-section of RD (rolling direction) -TD (transverse direction) was observed.

## Author Contributions

Z.F.Z. proposed the concepts of TWIP copper alloys and SISP tendency. R.L. designed the mechanical experiments and proposed the models and mechanisms. R.L., Z.J.Z., L.L.L., X.H.A. and Z.F.Z. analyzed the data and wrote the paper. Z.F.Z. revised the paper. All authors contributed to the scientific discussions.
